# Malignant melanoma of the penis and urethra: one case report

**DOI:** 10.1186/1477-7819-12-340

**Published:** 2014-11-11

**Authors:** Yunxiang Li, Haichao Yuan, Anguo Wang, Zongping Zhang, Ji Wu, Qiang Wei

**Affiliations:** Department of Urology, West China Hospital, Sichuan University, Guoxue Xiang #37, Chengdu, Sichuan P.R. China; Department of Urology, Nan Chong Central Hospital, Nanchong Renmin Nanlu #97, Nanchong, Sichuan 610041 P.R. China

**Keywords:** Melanoma, Penis, Urethra

## Abstract

We present a case of a patient with malignant melanoma of the glans penis and urethra, which was found in a 53-year-old man with nonhealing ulcerative penile lesion and bilateral, clinically palpable inguinal lymphadenopathies at diagnosis. A diagnostic biopsy showed the characteristics of a melanoma. We treated the patient with total penectomy and bilateral inguinal lymph node dissection. After surgery, chemotherapy with bleomycin, vincristine and cisplatin and immunotherapy with thymosin injection were started. No recurrence or metastasis occurred during the 3 years after the operation. Melanoma of the penis is very rare, and early diagnosis is important because the patient prognosis is very poor.

## Background

Malignant melanoma of the penis is an extremely rare malignancy that accounts for less than 2% of all primary penile malignant lesions [1]. They are located on the glans penis (55%), prepuce (28%), penile shaft (9%) and urethral meatus (8%) [2]. Malignant melanoma of the penis and urethra as comorbid presentations are more rare. We present a case of a patient with multifocal melanoma of both the glans penis and urethra, which, to the best of our knowledge, has not been previously described. We also provide a review of the literature, with emphasis on the pathogenesis and treatment of melanoma of the penis and urethra.

## Case presentation

**Figure 1:**
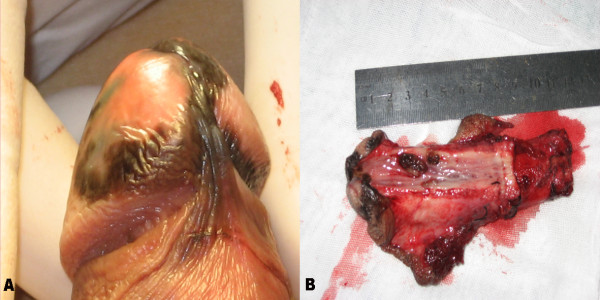
**Multifocal melanoma of both the glans penis and urethra. (A)** Uniform dark brown pigmented macule partially occupying the glans penis, coronary sulcus and urethral meatus. **(B)** Longitudinal incision of the penis showed 8-cm melanin pigmentation in the pendulous urethra.

**Figure 2:**
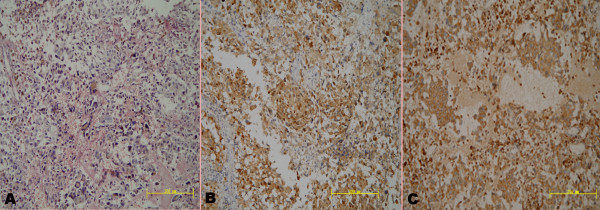
**Histologic specimens. (A)** Numerous atypical melanocytic cells with large hyperchromatic nuclei and abundant cytoplasm. The melanocytes were arranged in nests (hematoxylin and eosin stain; original magnification, ×200). **(B)** Sections of the main tumor mass show strong positive staining for HMB-45 (original magnification, ×200). **(C)** Sections of the main tumor mass show strong positive staining for CD68. The melanocytes were arranged in nests (original magnification, ×200).

A 53-year-old man presented to our institution in 2009 with a 24-month history of difficulty in urinating and a 6-year history of melanin pigmentation of the glans penis. He did not have a family history of malignant melanoma. He had experienced urethral orifice suffusion after sex during the preceding 4 months. His physical examination revealed patchy erythema melanin pigmentation that was scattered in the glans penis and coronary sulcus and involved the urethral meatus (Figure 1A). The melanin-like lesions were discovered in the urethra through urinary cystoscopy. We performed a punch biopsy in two different sites: the glans penis and urethra. His histopathological examination revealed a malignant melanoma. Computed tomographic scans of the head, chest and abdomen were negative for metastatic disease. There was no palpable inguinal lymphadenopathy. Total penectomy and bilateral inguinal lymph node dissection were performed. The penis was cut open longitudinally, and we found 8-cm melanin pigmentation in the pendulous urethra (Figure 1B). Pathological sections showed that the malignant melanoma invaded into superficial muscular layer. Histologic examination of these specimens revealed positive staining with hematoxylin (Figure 2A), HMB-45 (Figure 2B) and CD68 (Figure 2C). Bilateral inguinal lymph node specimens were negative. After surgery, the patient was started on an 8-week course of chemotherapy with bleomycin, vincristine and cisplatin and immunotherapy with thymosin injection. The patient had no tumor recurrence at the 2-year follow-up and remains alive to date.

## Discussion

Melanoma of the penis is rare [3]. It is a disease of the elderly, with a peak incidence in the 50- to 70-year-old age group. The peak incidence of cutaneous melanomas in other areas of the body occurs in patients 40 to 49 years of age [1, 4]. Because melanoma can metastasize to any tissue or organ early, it is one of the most dangerous tumors. The 2- and 5-year overall survival rates are 63% and 31%, respectively [4]. In several studies, researchers have reported melanoma of both the penis and the urethra. However, melanoma of the urethra is less rare than that of the penis. Involvement of the urethra occurs most frequently in the fossa navicularis. It occurs less often in the pendulous, bulbous and prostatic areas [5]. In our patient, melanomas were found in the penis and urethra at the same time.

Prediction of the clinical course of melanoma is based mainly on tumor thickness. However, the assessment of tumor thickness alone is not enough; other important factors in the prognosis are the tumor’s extent of involvement of local structures and whether there is clinical or histopathological evidence of metastases in the inguinal or pelvic lymph nodes [4]. Adverse prognostic factors are thickness (≥3.5 mm), ulceration and diameter (≥15 mm) [6].

Early diagnosis is of importance because the risk of distant metastases is high [7]. Melanoma is potentially curable if the pathological characteristics are favorable [8], but penile melanomas are usually diagnosed late. Clinically, they may vary in presentation from macules to papules and nodules, all of varying color. The absence of symptoms, the low level of public awareness and the difficulty associated with treatments at this site, as well as—not least in importance—embarrassment at being examined, all contribute to the delay in diagnosis [6]. Therefore, clinicians should be highly suspicious when examining any penile pathology. In clinical practice, we found that it was very difficult to recognize a pigmented penile lesion as a melanoma. Some authors have reported that dermoscopy is helpful for distinguishing a melanocytic lesion from a nonmelanocytic one and can play a role in establishing whether a melanocytic lesion is benign or malignant, which will improve the diagnosis [6, 9].

Staging of the disease, the choice of treatment and the patient’s prognosis have traditionally been based on a system developed by Bracken *et al*., with stage I disease confined to the penis, stage II being metastatic to the regional lymph nodes and stage III representing disseminated disease [10]. Treatment of melanoma of the glans penis and urethra is surgical, but the main area of controversy lies with the extent of surgery for localized disease. Some authors [10, 11] believe in an aggressive surgical approach with total amputation of the penis and bilateral ilioinguinal node dissection. Recently, however, most authors [12, 13] have recommended wide local excision or distal amputation without lymph node dissection for lesions. Their rationale is that radical surgery may be overtreatment because it carries no survival advantage for stage II or III patients.

The prognosis for patients with melanoma is poor because of the lack of effective systemic chemotherapy. Combination chemotherapy consisting of six cycles of dacarbazine, carmustine, cisplatin and tamoxifen gives the best results. An overall response rate of about 45% and a complete response rate of 12% to 14% have been reported. It is thought that 5% of patients with stage III melanoma can be cured [14, 15]. In one study [16], researchers reported that treatment with high doses of adjuvant interferon in patients with high-risk stage II and stage III melanoma reduced the risk of disease recurrence and increased the median disease-free survival. In our patient, we started adjuvant therapy with thymosin and bleomycin, vincristine and cisplatin, without interferon. The prognosis for our patient is very good, and he remains alive to date.

## Conclusions

We think that early detection was the key to effective treatment of our patient. In such patients, early recognition, early and appropriate aggressive surgical therapy and the development of effective adjuvant therapy are important elements of improving the prognosis.

## Consent

Written informed consent was obtained from the patient for publication of this case report and any accompanying images. A copy of the written consent is available for review by the Editor-in-Chief of this journal.
